# External validation of a prediction model for surgical site infection after thoracolumbar spine surgery in a Western European cohort

**DOI:** 10.1186/s13018-018-0821-2

**Published:** 2018-05-16

**Authors:** Daniël M. C. Janssen, Sander M. J. van Kuijk, Boudewijn B. d’Aumerie, Paul C. Willems

**Affiliations:** Department of Orthopaedic Surgery, Research School CAPHRI, Maastricht University Medical Center, P. Debyelaan 25, 6229 HX Maastricht, the Netherlands

**Keywords:** Spine surgery, Instrumentation, Surgical site infection, Prediction model, External validation

## Abstract

**Background:**

A prediction model for surgical site infection (SSI) after spine surgery was developed in 2014 by Lee et al. This model was developed to compute an individual estimate of the probability of SSI after spine surgery based on the patient’s comorbidity profile and invasiveness of surgery. Before any prediction model can be validly implemented in daily medical practice, it should be externally validated to assess how the prediction model performs in patients sampled independently from the derivation cohort.

**Methods:**

We included 898 consecutive patients who underwent instrumented thoracolumbar spine surgery.

To quantify overall performance using Nagelkerke’s *R*^2^ statistic, the discriminative ability was quantified as the area under the receiver operating characteristic curve (AUC). We computed the calibration slope of the calibration plot, to judge prediction accuracy.

**Results:**

Sixty patients developed an SSI. The overall performance of the prediction model in our population was poor: Nagelkerke’s *R*^2^ was 0.01. The AUC was 0.61 (95% confidence interval (CI) 0.54–0.68). The estimated slope of the calibration plot was 0.52.

**Conclusions:**

The previously published prediction model showed poor performance in our academic external validation cohort. To predict SSI after instrumented thoracolumbar spine surgery for the present population, a better fitting prediction model should be developed.

## Background

Surgical site infection (SSI) after spinal fusion can have devastating consequences and morbidity that may yield substantial physical limitations with a distinct decrease in quality of life and overall increased health care costs [1]. SSIs can be difficult both to diagnose and to treat. One or more operative debridements combined with prolonged antibiotic treatment may be necessary to eradicate the infection [1–4].

In spine surgery, a relatively high incidence of SSIs of up to 12% is observed, depending on diagnosis, surgical approach, the use of spinal instrumentation, and the complexity of the procedure [5–8]. Prior research identified several factors associated with an increased risk of SSI: advanced age, obesity, diabetes, smoking, malnutrition, and prolonged duration of surgery [5, 6, 9–11]. Most of these risk factors are quantified as relative risk or odds ratio. These values are difficult to use in clinical workup before operation to estimate the risk for postoperative SSI and personalize decision-making on individual patient characteristics.

A prediction model is an appropriate tool for shared decision-making during workup to evaluate the individual risk of SSI after spinal surgery and possibly to prevent SSI and its devastating consequences by taking measures before and during surgery [1]. Lee et al. developed a prediction model for SSI after spine surgery that was derived from a surgical spine register of the USA (the Spine End Results Registry). This model was developed to compute an individual estimate of the probability of SSI after spine surgery based on the patient’s comorbidity profile and invasiveness of surgery [11].

A prediction model is most valuable when it is generally applicable. However, before any prediction model can be validly implemented in daily medical practice, it should be externally validated to assess how the prediction model performs in patients sampled independently from the derivation cohort. To the best of our knowledge, the prediction model of Lee et al. has never been externally validated. The aim of the present study was to externally validate the prediction model by Lee et al. in a Western European cohort of patients who received instrumented thoracolumbar spine surgery.

## Methods

### Study population

For the external validation, we used the data from a prospective cohort of patients > 18 years who underwent instrumented spine surgery from January 1999 up to January 2016 in the Maastricht University Medical Centre.

All operations were performed by three experienced orthopedic surgeons specialized in spine surgery. In some cases, neurosurgeons participated in the operation. All patients underwent an instrumented posterior (posterolateral or interbody) spinal fusion of the thoracolumbar spine with or without an additional procedure (anterior fusion or release, spinal decompression, removal of instrumentation, tumor resection or (partial) corpectomy).

Patients were followed for a minimum of 1.5 year after the index operation to monitor all complications and outcomes of the procedure. All complications, extensive demographics, comorbidity, and surgical details were recorded by collecting data out of all electronic and paper records of the patients. For the preexisting medical comorbidities that were used in the prediction model of Lee et al. (congestive heart failure, diabetes, rheumatoid arthritis), we used the following definition:

Congestive heart failure—a proven decrease of ejection fraction of the heart on ultrasonography and all conditions that decrease the ejection fraction of the heart, including myocardial infarction, angina pectoris, and mitral valve disease in medical history

Diabetes mellitus—insulin-dependent and insulin-independent diabetes mellitus

Rheumatoid arthritis—rheumatoid arthritis, ankylosing spondylitis or psoriatic arthritis that had been officially diagnosed by a rheumatologist

We calculated the surgical invasiveness index (SII), as used by Lee et al. for all patients. This index is a validated instrument with a range from 0 to 48 points and contains the sum of six weighted surgical components: number of levels anterior decompressed, anterior fused, anterior instrumented, posterior decompressed, posterior fused, and posterior instrumented. The weight for each component represents the number of vertebral levels at which each respective component has been performed [12].

The primary outcome of interest was SSI. The diagnosis of surgical site infection in our patient cohort was based on the CDC (Centre for Disease Control and prevention) criteria [13] and the Dutch national PREZIES (*prevention of hospital infections through surveillance*) network [14]. An SSI was considered to be deep if it presented at the site of the operation with involvement of the subfascial tissues. This definition is independent of return to the operating room for irrigation and debridement, in contrast to the definition of SSI used by Lee et al. who defined SSI as an infection requiring return to the operating room. We included all deep infections, even those we did not treat with a re-operation because of terminal illness. All patients had an outpatient appointment at 1 year after the index operation to be registered as “SSI” or “No SSI.”

### Statistical analysis

For predictor variables that were incomplete, we used stochastic regression imputation. This ensures all observed data can be used for the analysis, preventing a potentially considerable loss of statistical precision. We used predictive mean matching to draw the values to be imputed.

### Prediction model

The prediction model of Lee et al. was based on the data of the Spine End Results Registry (SERR). This is a prospectively collected registry for all surgical spine patients at the University of Washington and Harborview Medical Center who underwent surgery from January 1, 2003, to December 31, 2004. This cohort included 1745 patients. One thousand five hundred thirty-two patients were included and were followed for adverse events. Seven hundred thirty-eight (48%) patients consented to provide detailed questionnaires of their risk factors. In 794 (52%) patients, some information about their risk factors, such as smoking status and alcohol use, were missing, as the data for these patients were found either by notification from hospital staff or by medical record review.

The prediction model consisted of seven predictor variables, i.e., body mass index (BMI) classified as normal (18.5 ≤ BMI < 25.0), underweight (BMI < 18.5), overweight (25.0 ≤ BMI < 30.0), and obese (BMI ≥ 30) (in the original article, it was not clear whether a BMI of 30.0 would classify as overweight or obese, so we included it in the obese range), diagnosis group (degenerative, trauma, or other), SII score, congestive heart failure (yes or no), diabetes (yes or no), rheumatoid arthritis (yes or no), and age.

In order to derive the prediction formula, we needed regression coefficients, including the intercept. These parameters were not published in the manuscript nor could they be retrieved from the website, or from the authors. Therefore, we took the natural logarithm of the odds ratios presented in the manuscript. These can be used to compute a risk score that ranks patients according to their risk but that does not yield the probability of an SSI. In addition, we used our own cohort to estimate the intercept so that the average predicted probability is exactly the same as the frequency of SSI. After obtaining all regression coefficients, including the intercept, we computed each individual’s probability of an SSI using the standard logistic regression formula.

### Prediction model performance

We quantified the external validity of the prediction model by computing measures of overall performance, discriminative ability, and calibration. To quantify overall performance, we computed Nagelkerke’s *R*^2^ statistic. Nagelkerke’s *R*^2^ is a pseudo-*R*^2^ measure for binary outcomes.

The prediction models’ discriminative ability was quantified as the area under the receiver operating characteristic (ROC) curve (AUC). It can be interpreted as the proportion of randomly drawn pairs in which the one developing an SSI has a higher predicted probability than the individual not developing an SSI. It can range between 0.5 and 1.0. The higher, the better the prediction model’s discriminative ability. As a sensitivity analysis, we computed the AUC on our sample after excluding deep infections that we did not treat with a re-operation as they would not have been regarded as events according to the definition in the study by Lee et al.

Calibration refers to the agreement between predicted and observed probabilities. We visually inspected the calibration plot to assess whether the prediction model over- or underestimates actual risk for certain risk-based subgroups and computed the calibration slope which ideally should be 1.

## Results

The cohort was comprised of a total of 949 patients. Fifty-one patients were excluded: 9 patients were diagnosed before the index operation with an infection after previous back surgery and 42 patients were excluded because there is too little information to be imputed. We included a total of 898 participants for the external validation, of whom 60 (6.7%) were subsequently diagnosed with an SSI, including two deep infections not treated with a re-operation because of terminal illness. Table 1 shows baseline characteristics of all patients included in the study. The predictor variable with the highest number of missing values in our dataset before imputation was BMI (52 missing, or 5.7%). All other predictor variables were completely observed. After imputation, all records could be used for the analysis.

**Table 1:** Baseline characteristics of all patients included in the study

Variable	All patients (898)	No SSI (838)	SSI (60)	Lee et al. (1532)
Age	52.2 (SD 16.1)	51.9 (SD 16.0)	56.9 (SD 16.5)	49.5
Gender	M 48.9%; F 51.1%	M 48.6%; F 51.4%	M 53.3%; F 46.7%	M 57%; F 43%
BMI	26.1 (SD 4.7)	26.0 (SD 4.5)	27.9 (SD 5.9)	27.7
ASA	1: 310 (34.5%)	1: 295 (35.2%)	1: 15 (25%)	
	2: 435 (48.4%)	2: 416 (49.6%)	2: 19 (31.7%)	
	3: 150 (16.7%)	3: 125 (14.9%)	3: 25 (41.7%)	
	4: 3 (0.3%)	4: 2 (0.2%)	4: 1 (1.7%)	
Diagnosis*	Trauma 199 (22.1%) De novo degenerative scoliosis 54 (6.0%) Adult spinal deformity 59 (6.5%) Degenerative spinal cord compression disorder 379 (42.1%) Malignancy 42 (4.7%) Failed back surgery 96 (10.7%) One- or two-level degenerative disorder of the spine 61 (6.8%) Spondylodiscitis 8 (0.9%)	Trauma 181 (21.6%) De novo degenerative scoliosis 51 (6.1%) Adult spinal deformity 58 (6.9%) Degenerative spinal cord compression disorder 358 (42.7%) Malignancy 35 (4.2%) Failed back surgery 90 (10.7%) One- or two-level degenerative disorder of the spine 58 (6.9%) Spondylodiscitis 7 (0.8%)	Trauma 18 (30.0%) De novo degenerative scoliosis 3 (5.0%) Adult spinal deformity 1 (1.7%) Degenerative spinal cord compression disorder 21 (35.0%) Malignancy 7 (11.7%) Failed back surgery 6 (10.0%) One- or two-level degenerative disorder of the spine 3 (5.0%) Spondylodiscitis 1 (1.7%)	Trauma 24.3% Degenerative 64.7%
SI score	10.3 (SD 5.9)	10.3 (SD 6.0)	10.1 (SD 5.1)	Mean 8.5
CHF	49 (5.5%)	44 (5.3%)	5 (8.3%)	
Diabetes	73 (8.2%)	66 (7.9%)	7 (11.6%)	
RA	20 (2.2%)	17 (2.0%)	3 (5.0%)	
Previous operation	253 (28.2%)	234 (27.9%)	19 (31.7%)	
Blood loss	1124 mL (SD 1201 mL)	1113 mL (SD 1211 mL)	1276 mL (SD 1044 mL)	
Surgical time	248 min (SD 100 min)	247 min (SD 99 min)	264 min (SD 123 min)	
Cage	42.0%	42.7%	32.7%	
Number of levels fused	3.2 (SD 2.9)	3.2 (SD 2.9)	3.3 (SD 2.5)	
Dural tear	91 (10.1%)	82 (9.8%)	9 (15.0%)	
Combined anterior approach Posterior approach	2.8% 97.2%	2.8% 97.2%	3.4% 96.6%	22.8% 58.7%
Smoking	285 (31.7%)	265 (31.6%)	20 (33.4%)	
Alcohol	334 (37.2%)	305 (36.4%)	29 (40.0%)	
Transfusion	281 (32.9%)	257 (32.2%)	24 (42.9%)	
Using NSAIDs post-OK	433 (48.2%)	398 (47.5%)	35 (58.3%)	
Using NSAID pre-OK	225 (25.1%)	205 (24.5%)	20 (33.3%)	
Amount of transfusion	279 mL (SD 675 mL)	273 mL (SD 682 mL)	367 mL (572 mL)	
Timing AB prophylaxis before surgery	37 min (SD 20 min)	37 min (SD 19 min)	42 min (SD 22 min)	
Mean FiO_2_ during surgery	48.9 (SD 12)	48.8 (SD 12)	49.6 (SD 14.4)	

*Degenerative spinal cord compression disorder = spondylolisthesis, spinal stenosis, HNP; De novo degenerative scoliosis = degenerative scoliosis, junctional kyphosis; Adult spinal deformity = kyphosis, juvenile scoliosis, adolescent scoliosis, neuromuscular scoliosis, idiopathic scoliosis; One- or two-level degenerative disorder of the spine = degenerative discopathy, spondylosis, facetarthrosis, adjacent segment degeneration; Fracture = fracture with and without myelum compression; Failed back surgery = failed previous total disc replacement, pseudoarthrosis, failed previous laminectomy, failed previous posterior fusion, failed previous discectomy, failed previous anterior fusion, hardware failure

The back-transforming of the odds ratios published by Lee et al. and the estimation of the intercept based on the present cohort yielded the following formula for the prediction of the probability of an SSI after spinal surgery:

Probability of SSI after spinal surgery = 1/(1 + e^−LP^), in which LP = − 3.73 + 1.12*CHF + 0.74*diabetes + 0.70*rheumatoid arthritis + 0.06*SII + 0.002*age + 0.48*trauma − 0.09*other + 0.79*underweight − 0.14*overweight + 0.34*obese.

For example, the probability to develop an SSI after spinal surgery for a 65-year-old overweight male, who has no comorbidities, who will be operated upon due to trauma, and who has an SII score of 10:

LP = − 3.73 + 1.12*0 + 0.74*0 + 0.70*0 + 0.06*10 + 0.002*65 + 0.48*1 − 0.09*0 + 0.79*0 − 0.14*1 + 0.34*0 = − 2.66. Hence, the probability of SSI after spinal surgery = 1/(1 + e^+ 2.56^) = 0.065 = 6.5%.

### Prediction model performance

**Fig. 1:**
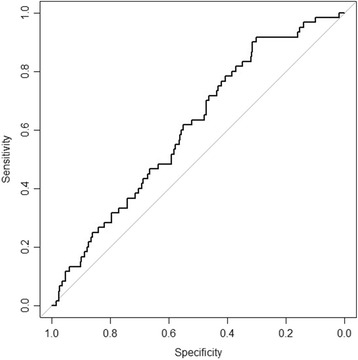
ROC curve of the prediction model by Lee et al. used to predict SSI

This model was subsequently externally validated. The overall performance was poor: Nagelkerke’s *R*^2^ was only 0.01, indicating poor predictive strength. The AUC of the model by Lee et al. applied to our cohort was 0.61 (95% confidence interval (CI) 0.54–0.68), indicating only mediocre discriminative ability (see Fig. 1). Only two patients had a deep infection but were not subsequently re-operated because of terminal illness. In the sensitivity analysis in which we excluded them from the analysis, the AUC did not differ substantially; the AUC was 0.62 (95% CI, 0.55–0.69).

**Fig. 2:**
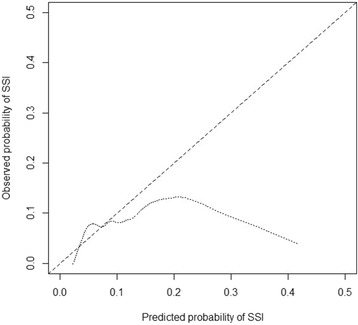
Calibration plot of the prediction model by Lee et al. used to predict SSI

The calibration plot is shown in Fig. 2. The risks of patients at high risk (say, 20% or higher) are on average severely overestimated, as indicated by the fact that the curve lies far beneath the 45° line of perfect calibration. For example, of all patients who had an estimated probability of SSI of about 30%, only 10% actually developed SSI. The estimated slope of the calibration plot was 0.52 compared to an ideal value of 1.

## Discussion

We externally validated a previously published prediction model for SSI after spine surgery after back-transforming the published ORs and estimating an intercept specific for our site. The prediction model performed poorly on overall fit, discriminative ability, and calibration. Often, previously developed models perform worse than expected on future patients, especially on patients from different settings. One explanation could be that there is a significant difference in the rate of SSI between our cohort (6.7%) and the cohort of Lee et al. (4.3%), which may have been caused by a difference in patient population. In contrast to the cohort of Lee et al., we solely included “instrumented” spinal procedures that are known to have a higher infection rate, as seen in the literature [15]. Lee et al. included patients of the Spine End Results Registry (SERR). In this database also, patients without instrumentation were included [16]. The average SI score in our sample was 1.8 points higher compared to the sample of Lee et al. Probably our procedures were more invasive because we solely included “instrumented” procedures and more long-trajectory fusion procedures (e.g., scoliosis). Cizik et al. concluded that surgical invasiveness is the strongest risk factor for SSI after spine surgery, even after adjusting for medical comorbidities, age, and other known risk factors [16]. Lee et al. included a higher percentage of men (57%) than we did in our population (49%). It has been reported that female sex is a predictor of surgical site infection after spine surgery [17, 18]. The mean age was approximately the same (49.5 vs. 52.2 years; SD 16.1) between the two populations just as the mean body mass index (27.7 vs. 26.1, SD 4.7). Also, the diagnosis for the index operation is more or less the same. 54.9% in our population had a degenerative condition for treatment (de novo degenerative scoliosis, degenerative spinal cord compression disorder, or one- or two-level degenerative lumbar disc disease) followed by 22.1% trauma, as compared to 64.7 and 24.3% of the population of Lee et al., respectively. All operations were performed using a posterior approach and in 2.8% combined with an anterior approach. This is in contrast to the population of Lee et al., where in 58.7% a posterior approach was used and in 22.8% a combined approach.

In both studies, there is the possibility of underdiagnosis of surgical site infection because of patients that may have been treated elsewhere for SSI without recording in the database. In our study, this would have been only possible in cases with an SSI more than 1 year after the index operation, because we registered the infection status of all patients at 1 year follow-up on the outpatient clinic.

A limitation of this external validation is the potential lack of similarity of definitions of predictor variables. Despite several mail attempts by our study group, the authors of the prediction model were not able to inform us about their methods. In addition, the incidence of preexisting medical comorbidities as used in the prediction model of Lee et al. (congestive heart failure, rheumatoid arthritis, and diabetes) could not be compared because these were not further specified in the article. A second limitation is the sample size of our cohort. Even though the absolute size is quite large, the number of events (SSI) is only 60. A study suggests using at least 100 events and 100 non-events for an external validation study [19]. Therefore, our results may be less precise.

In prior research, more risk factors were identified to increase the risk of SSI after spine surgery than used in the prediction model of Lee et al. In our opinion, some of these factors would be important to include in a model for SSI following (instrumented) spinal surgery of the thoracolumbar spine: smoking, alcohol use, and previous spine surgery [5, 6, 20]. These factors are important in shared decision-making and communication with patients undergoing spinal surgery because some of these factors, such as smoking behavior, can be adapted during workup.

## Conclusion

The model presented by Lee et al. shows poor predictive performance in our cohort of Western European patients undergoing instrumented spinal surgery. For valid and accurate prediction of SSI after instrumented spine surgery in an academic center, a better prediction model should be developed, preferably with more, and better defined risk factors earlier described in literature for a patient population that is better comparable with the population in our academic spine center. After the development of such a prediction model, this should also be externally validated in similar populations to use it as a broad and more general model. A valuable tool for validations of new models could be high-volume national and international registry data to compare factors such as diagnosis, operations, comorbidity, and incidence of infection in large patient populations, because of the low incidence of SSI in spine surgery.

## Abbreviations

AUC: Area under the receiver operating characteristic curve
BMI: Body mass index
CDC: Centre for disease control and prevention
CHF: Congestive heart failure
LP: Linear prediction
METC: Medical ethics committee
OR: Odds ratio
PREZIES: Prevention of hospital infections through surveillance
ROC: Receiver operating characteristic
SD: Standard deviation
SERR: The Spine End Results Registry
SII: Surgical invasiveness index
SSI: Surgical site infection
USA: United States of America
WMO: Medical Research Involving Human Subjects Act
